# Harnessing the Power of Water: A Scoping Review of Hydrokinesiotherapy as a Game-Changer in Knee Osteoarthritis Management

**DOI:** 10.3390/jcm13195811

**Published:** 2024-09-28

**Authors:** Roberto Tedeschi, Federica Giorgi, Danilo Donati

**Affiliations:** 1 Department of Biomedical and Neuromotor Sciences, Alma Mater Studiorum, University of Bologna, Via Zamboni 33, 40126 Bologna, Italy; 2 Pediatric Physical Medicine and Rehabilitation Unit, IRCCS Institute of Neurological Sciences, 40124 Bologna, Italy; federica.giorgi15@gmail.com; 3 Physical Therapy and Rehabilitation Unit, Policlinico di Modena, 41121 Modena, Italy; danilo.donati@unimore.it; 4 Clinical and Experimental Medicine PhD Program, University of Modena and Reggio Emilia, 41121 Modena, Italy

**Keywords:** hydrokinesiotherapy, knee osteoarthritis, pain management, physical function, aquatic therapy

## Abstract

**Background:** Knee osteoarthritis (OA) is a prevalent condition that significantly impairs pain, mobility, and quality of life. Hydrokinesiotherapy, a water-based exercise therapy, is gaining traction as a potential alternative to traditional land-based rehabilitation for managing knee OA symptoms. **Methods:** This scoping review aimed to evaluate the effectiveness of hydrokinesiotherapy compared to standard land-based physical therapy and self-administered exercise regimens in improving pain, joint mobility, quality of life, and physical function in patients with knee OA. Five randomized controlled trials (RCTs) were included, assessing outcomes using measures such as the WOMAC score, Visual Analog Scale (VAS), and SF-12. The studies were critically appraised using the PEDro scale and the RoB-2 tool to determine the quality and risk of bias. **Results:** Hydrokinesiotherapy consistently demonstrated superior outcomes in pain reduction, joint mobility, and physical function across all studies. Patients in the hydrokinesiotherapy groups reported significant improvements in pain and mobility, which were strongly associated with enhanced quality of life. The review also highlighted the potential for hydrokinesiotherapy to serve as an effective alternative or complement to land-based exercises, particularly in populations experiencing severe symptoms. **Conclusions:** Hydrokinesiotherapy is an effective intervention for managing knee OA, offering significant improvements in key clinical outcomes. Given its benefits, hydrokinesiotherapy should be considered a valuable addition to knee OA treatment protocols. However, further research is needed to confirm long-term effects and to explore ways to improve accessibility to this therapeutic option.

## 1. Introduction

Knee osteoarthritis (OA) is a progressive and chronic disease often leading to significant morbidity and decreased quality of life. While conventional therapies for knee OA, such as pharmacological and land-based physical therapy, are widely accepted, there are notable gaps in understanding the long-term benefits and comparative effectiveness of alternative treatments like hydrokinesiotherapy. Hydrokinesiotherapy has gained interest due to its ability to reduce joint loading, potentially benefiting patients who struggle with land-based exercises. However, questions remain regarding its optimal application, long-term effects, and effectiveness compared to other rehabilitation strategies. This scoping review aims to map the current evidence and identify areas that require further research. Conservative management encompasses a range of non-invasive therapies, with hydrokinesiotherapy being a prominent modality. Hydrokinesiotherapy leverages the physical properties of water, such as buoyancy, resistance to movement, and hydrostatic pressure, for rehabilitative purposes. Knee osteoarthritis (OA) is recognized as the most prevalent form of osteoarthritis; according to recent estimates, the prevalence of knee OA is approximately 28% in individuals over the age of 45, and its incidence is projected to rise with the aging population [1,2,3,4]. Pharmacological treatments primarily focus on anti-inflammatory medications, including corticosteroids, to manage pain [5,6,7,8] and inflammation [9,10,11,12,13]. Conservative management encompasses a range of non-invasive therapies, with hydrotherapy [14], specifically hydrokinesiotherapy, being a prominent modality. Hydrokinesiotherapy leverages the physical properties of water, such as buoyancy, resistance to movement, and hydrostatic pressure, for rehabilitative purposes [15,16,17,18,19,20,21]. The warm water used in hydrokinesiotherapy facilitates muscle relaxation, which, in turn, reduces pain during both passive and active joint mobilization [14,22,23,24,25]. Immersion in water naturally slows down movement patterns, allowing for a more controlled assessment of movement effectiveness, correction of compensatory mechanisms, and relearning of proper movement patterns [26,27,28]. The therapeutic benefits of water are particularly relevant in the treatment of knee OA [29]. Buoyancy, as described by Archimedes’ principle, reduces the effective weight of the body, thereby decreasing the load on the joints during exercise [30,31,32,33,34,35,36]. This reduction in joint loading is particularly beneficial for individuals with knee OA, as it allows for the performance of weight-bearing exercises even in the early stages of rehabilitation without exacerbating joint pain or damage. Water resistance, which opposes movement, provides a natural form of resistance training that can be adjusted simply by varying the speed of movement [37,38,39]. Additionally, hydrostatic pressure, which increases with the depth of immersion, exerts a uniform force on the submerged body, promoting venous and lymphatic return, and potentially reducing joint edema and increasing cardiac output [40]. Hydrokinesiotherapy is widely applicable across various fields of rehabilitation, including orthopedics [41], neurology [42], and pediatrics. However, there are also several general and specific contraindications to water-based therapy, such as uncompensated cardiac conditions, severe respiratory disorders, uncontrolled hypertension or hypotension, active dermatological conditions, open wounds, severe urinary or fecal incontinence, and certain neurological conditions like uncontrolled epilepsy [43,44]. However, despite the growing body of evidence supporting hydrokinesiotherapy, there remain significant gaps in our understanding of its long-term benefits, optimal treatment duration, and its efficacy compared to conventional land-based therapies. These gaps necessitate a thorough review of existing evidence to guide future clinical practice. It is essential to note that while hydrokinesiotherapy offers numerous advantages, its applicability may be limited by certain contraindications, such as severe cardiac or respiratory conditions, active skin infections, or uncontrolled hypertension. These limitations underscore the need for careful patient selection to ensure safety and maximize therapeutic benefits. Despite the growing interest in hydrokinesiotherapy, there remain significant gaps in understanding its long-term benefits, optimal treatment duration, and effectiveness across diverse patient populations. A scoping review is particularly suited to map the breadth of existing literature on hydrokinesiotherapy and identify areas where further research is needed. This methodology allows for the inclusion of a wide range of study types, providing a comprehensive overview of the current evidence. The primary objective of this systematic review is to evaluate the effectiveness of hydrokinesiotherapy in managing knee OA, particularly in comparison to standard land-based physical therapy performed under the supervision of a physiotherapist or self-administered exercise regimens undertaken by patients at home. A scoping review methodology was selected because it allows for a comprehensive exploration of the evidence on hydrokinesiotherapy, while focusing on randomized controlled trials (RCTs). This approach is particularly suited for identifying research gaps, summarizing the range of existing evidence, and mapping future research directions in a rapidly growing field such as aquatic therapy for knee OA. To achieve this objective, the review focuses on randomized controlled trials (RCTs) that specifically address the efficacy of hydrokinesiotherapy relative to these standard treatment modalities. The outcomes of interest include improvements in pain, joint mobility, quality of life, physical function, and overall patient satisfaction. Through this review, we aim to provide evidence-based recommendations for the use of hydrokinesiotherapy as a viable and potentially superior treatment option for patients suffering from knee osteoarthritis.

## 2. Methods

The present scoping review was carried out in accordance with the JBI methodology [45] for scoping reviews, and the reporting was guided by the Preferred Reporting Items for Systematic Reviews and Meta-Analyses extension for Scoping Reviews (PRISMA-ScR) checklist [46].

### 2.1. Review Question

We formulated the following research question: “Is hydrokinesiotherapy more effective than standard land-based physical therapy or self-administered exercise regimens in improving pain, joint mobility, quality of life, and physical function in patients with knee osteoarthritis?”

### 2.2. Eligibility Criteria

Studies qualified for inclusion if they adhered to the following Population, Concept, and Context (PCC) framework criteria. The PCC framework used in this review follows the guidelines outlined by the Joanna Briggs Institute for conducting scoping reviews:

Population (P): The study participants were adults aged 45 years and older with a clinical and/or radiographic diagnosis of knee osteoarthritis (OA). Participants included in the studies should not have undergone knee joint replacement surgery and should be independent in mobility, allowing them to access rehabilitation centers without assistance.

Concept (C): Any form of yoga intervention where the concept of interest was the effectiveness of hydrokinesiotherapy as a treatment modality for knee osteoarthritis. The studies needed to compare hydrokinesiotherapy with standard land-based physical therapy or self-administered exercise regimens. The focus was on evaluating key outcomes such as pain reduction, improvement in joint mobility, enhancement of physical function, and overall quality of life.

Context (C): The context of the studies included any setting where hydrokinesiotherapy was performed, such as rehabilitation centers, clinics, or hospitals. The studies could be conducted in various geographic locations and should provide a comparison to standard treatments commonly used in the management of knee osteoarthritis, including physical therapy in a gym or home-based exercise programs. The review included studies published in peer-reviewed journals, written in English, and conducted from the year 2000 onwards.

### 2.3. Exclusion Criteria

Studies that failed to meet the specified PCC criteria were excluded.

### 2.4. Search Strategy

A preliminary search of MEDLINE via the PubMed interface was conducted to locate relevant articles, and the index terms associated with those articles were utilized to create a comprehensive search strategy for MEDLINE. This search strategy, incorporating all identified keywords and index terms, was further modified for use in Cochrane Central, Scopus, PEDro, and Web of Science. Additionally, gray literature and the reference lists of pertinent studies were reviewed. The searches took place on 31 July 2024, without any date restrictions.

PubMed: (“Hydrotherapy” [MeSH Terms] OR “Aquatic Therapy” [MeSH Terms] OR “Aquatic Exercise” [MeSH Terms] OR “Hydrokinesiotherapy” OR “aquatic therapy”) AND (“Osteoarthritis, Knee” [MeSH Terms] OR “knee osteoarthritis” OR “osteoarthritis of the knee” OR “knee OA”) AND (“Randomized Controlled Trial” [Publication Type] OR “RCT” [All Fields])

Scopus: (“Hydrotherapy” OR “Aquatic Exercise” OR “Hydrokinesiotherapy”) AND TITLE-ABS-KEY(“knee osteoarthritis” OR “osteoarthritis of the knee” OR “knee OA”) AND TITLE-ABS-KEY(“randomized controlled trial” OR “RCT”)

Cochrane: (“Hydrotherapy” OR “Aquatic Exercise” OR “Hydrokinesiotherapy”) AND (“Knee Osteoarthritis” OR “Osteoarthritis of the Knee” OR “Knee OA”).

Web of Science: TS = (“Hydrotherapy” OR “Aquatic Exercise” OR “Hydrokinesiotherapy”) AND TS = (“Knee Osteoarthritis” OR “Osteoarthritis of the Knee” OR “Knee OA”) AND TS = (“randomized controlled trial” OR “RCT”)

Pedro: (hydrotherapy OR “aquatic exercise” OR hydrokinesiotherapy) AND (“knee osteoarthritis” OR “osteoarthritis of the knee” OR “knee OA”) AND (randomized controlled trial OR RCT).

### 2.5. Study Selection

The outlined process followed a systematic approach to study selection for a scoping review. First, search results were gathered and organized using Zotero (7.0.3), with duplicates eliminated. The screening process was conducted in two stages: an initial review of titles and abstracts followed by a full-text evaluation. Both stages were independently completed by two authors, with a third author resolving any disagreements. The selection process adhered to the PRISMA 2020 guidelines to ensure transparency and accuracy. This thorough methodology aimed to identify relevant articles that directly address the research question, ensuring a comprehensive and systematic review process.

### 2.6. Data Extraction and Data Synthesis

Data extraction for the scoping review was performed using a form based on the JBI tool, capturing essential information such as authorship, publication country and year, study design, patient characteristics, outcomes, interventions, procedures, and other relevant details. Descriptive analyses of the extracted data were carried out, with results presented in numerical format to illustrate the distribution of studies. The review process was transparently documented, and the data were summarized in tables to facilitate the easy comparison and comprehension of the key aspects and findings of the included studies.

## 3. Results

**Figure 1:**
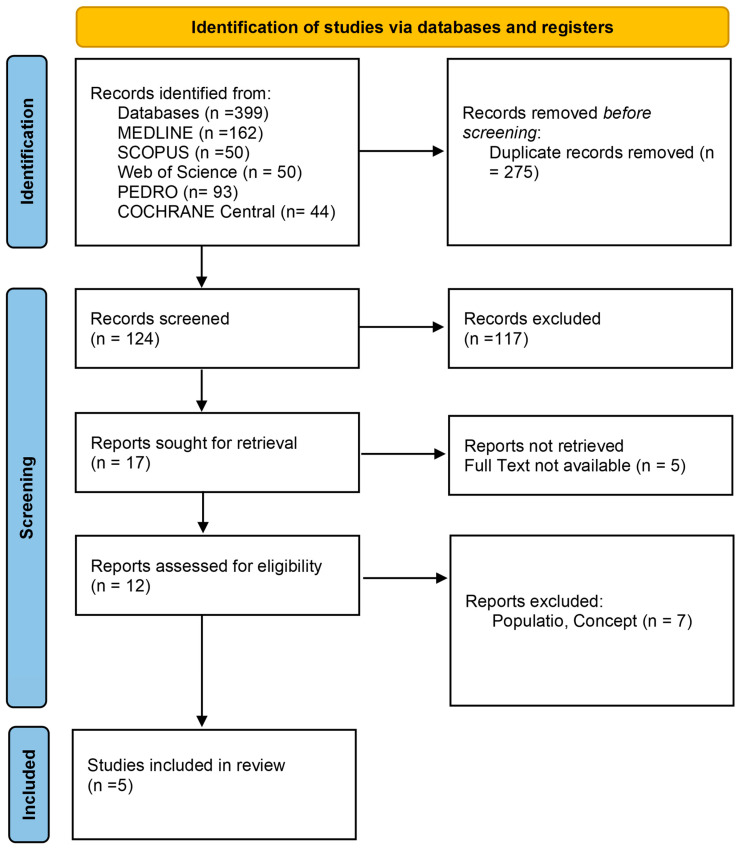
Preferred reporting items for systematic reviews and meta-analyses 2020 (PRISMA) flow-diagram.

As illustrated in the PRISMA 2020 flow diagram (Figure 1), out of 399 records identified through the initial literature searches, 394 were excluded, leaving 5 articles for inclusion (Table 1, Table 2 and Table 3). The quality of the included studies was evaluated using the PEDro scale (Table 4) and the ROB2 tool (Table 4). The PEDro scale and the RoB-2 tool were applied following established guidelines for assessing the methodological quality and risk of bias in randomized controlled trials.

### 3.1. Pain Reduction

Hydrokinesiotherapy was found to be consistently effective in reducing pain among patients with knee osteoarthritis across all studies.

- Foley et al. (2003) [22] demonstrated that both hydrotherapy and gym-based exercise interventions resulted in significant pain reduction compared to the control group. However, no statistically significant difference was noted between hydrotherapy and gym-based exercise, indicating that both modalities are comparably effective in alleviating pain.
- Azizi et al. (2019) [14] reported a significant decrease in pain levels in the aquatic exercise group, as measured by the Visual Analog Scale (VAS), compared to the control group. The reduction in pain was strongly correlated with improvements in balance, suggesting a multifactorial benefit of hydrokinesiotherapy.
- Taglietti et al. (2018) [23] found that participants in the aquatic exercise group exhibited significant improvements in WOMAC pain scores relative to the patient education group, highlighting the superior efficacy of hydrokinesiotherapy in pain management.
- Dias et al. (2017) [24] also confirmed that hydrokinesiotherapy led to significant pain reduction, as assessed by the WOMAC pain subscale, demonstrating its effectiveness in an older female population.
- Khruakhorn et al. (2021) [25] showed that both hydrotherapy and land-based exercises reduced pain, but the hydrotherapy group experienced a more substantial reduction, contributing to better overall outcomes in mobility and quality of life.

### 3.2. Joint Mobility

Improvements in joint mobility were a prominent outcome associated with hydrokinesiotherapy.

- Foley et al. (2003) [22] observed that both hydrotherapy and gym-based exercises enhanced joint mobility, with no significant difference between the two, as measured by step kinematic parameters. This indicates that hydrokinesiotherapy is as effective as traditional exercise in improving joint mobility.
- Azizi et al. (2019) [14] demonstrated significant improvements in knee range of motion (ROM) in the aquatic exercise group, which were associated with reduced pain and better balance, indicating that hydrokinesiotherapy effectively enhances joint flexibility and function.
- Taglietti et al. (2018) [23] reported significant reductions in joint stiffness, as evidenced by improvements in the WOMAC stiffness subscale among participants in the aquatic exercise group, reflecting increased ease of movement.
- Dias et al. (2017) [24], although primarily focused on pain and functional outcomes, noted improvements in knee flexion and extension in the hydrotherapy group, suggesting enhanced joint mobility.
- Khruakhorn et al. (2021) [25] found that joint mobility, assessed through dynamic movements like the time up and go (TUG) test and the stair climb test (SCT), improved in both intervention groups, with slightly better outcomes in the hydrotherapy group.

### 3.3. Quality of Life

Hydrokinesiotherapy was associated with significant improvements in quality of life across multiple studies.

- Foley et al. (2003) [22] reported significant improvements in the SF-12 physical component score in the hydrotherapy group, indicating better health-related quality of life, although the gym-based exercise group showed more pronounced improvements in mental health.
- Azizi et al. (2019) [14] documented notable enhancements in quality of life within the aquatic exercise group, driven by pain reduction and improved joint mobility, which contributed to better overall health and well-being.
- Taglietti et al. (2018) [23] observed significant gains in quality of life, particularly in physical health domains, among participants in the aquatic exercise group, as opposed to the patient education group, underscoring the holistic benefits of hydrokinesiotherapy.
- Dias et al. (2017) [24] also highlighted significant improvements in quality of life, particularly in the physical function and pain domains, among women undergoing hydrokinesiotherapy.
- Khruakhorn et al. (2021) [25] reported that both hydrotherapy and land-based exercises led to improvements in quality of life, with the hydrotherapy group showing slightly superior results, particularly in the WHOQOL-BREF-THAI physical health domain.

### 3.4. Physical Function

Hydrokinesiotherapy significantly enhanced physical function across the studies.

- Foley et al. (2003) [22] found that physical function, including muscle strength and walking speed, improved in both the hydrotherapy and gym-based exercise groups. The improvements were comparable, highlighting the effectiveness of hydrokinesiotherapy in enhancing functional capacity.
- Azizi et al. (2019) [14] demonstrated significant improvements in physical function in the aquatic exercise group, particularly in tasks involving balance and coordination, which were closely linked to pain reduction and joint mobility.
- Taglietti et al. (2018) [23] reported significant improvements in the functional abilities of participants in the aquatic exercise group, as measured by the WOMAC functional subscale. These improvements facilitated better performance in daily activities.
- Dias et al. (2017) [24] showed significant gains in muscle strength and endurance in the hydrotherapy group, which were assessed through isokinetic testing and other performance measures, confirming the physical benefits of hydrokinesiotherapy.
- Khruakhorn et al. (2021) [25] observed improvements in mobility and strength, particularly in the hydrotherapy group, as evidenced by significant results in the TUG and SCT outcomes, suggesting that hydrokinesiotherapy may be slightly more effective than land-based exercises in improving physical function.

### 3.5. Summary

Hydrokinesiotherapy consistently demonstrated superior efficacy in improving pain, joint mobility, quality of life, and physical function in patients with knee osteoarthritis, often outperforming standard land-based physical therapy and patient education. These findings reinforce the role of hydrokinesiotherapy as a valuable intervention for managing knee osteoarthritis, offering comprehensive benefits across multiple critical outcomes.

## 4. Discussion

This scoping review highlights the breadth of evidence supporting the use of hydrokinesiotherapy for knee OA, with consistent reports of improvements in pain, mobility, and quality of life. However, this evidence is varied in terms of long-term outcomes and treatment protocols. There is a need for further research to explore gaps related to the sustainability of benefits and the integration of land-based exercises following aquatic therapy. When compared to traditional land-based physical therapy and self-administered exercise regimens, hydrokinesiotherapy consistently demonstrated superior outcomes across multiple domains, including pain reduction, joint mobility, quality of life, and physical function. These results align with the growing body of evidence supporting the therapeutic benefits of aquatic-based exercises, particularly in populations with musculoskeletal disorders [47,48,49]. Pain management remains a central objective in the treatment of knee OA, and the observed reductions in pain across studies suggest that hydrokinesiotherapy is an effective modality in this regard. The immersion in water likely plays a crucial role in mitigating pain through several mechanisms. The buoyancy provided by water reduces the gravitational load on joints, thereby decreasing compressive forces and alleviating discomfort. Additionally, the warmth of the water may enhance blood circulation and relax muscles, further contributing to pain relief. It is important to note that while land-based exercises also effectively reduce pain, the unique properties of water might offer additional benefits, particularly for patients who experience significant pain during weight-bearing activities on land [14,22]. However, despite the positive outcomes, the variability in pain reduction among studies suggests that individual factors, such as the severity of OA and baseline functional status, may influence the extent of pain relief experienced by patients. This variability underscores the importance of personalized treatment approaches that consider patient-specific characteristics when implementing hydrokinesiotherapy [23,24,25]. The enhancement of joint mobility observed in the studies reviewed is a critical outcome, as restricted mobility is a common and debilitating feature of knee OA. Clinicians should consider transitioning patients from water-based to land-based exercises based on individual progress in pain reduction, joint mobility, and functional capacity. Ideally, this transition could begin when patients exhibit sufficient improvement in aquatic environments, allowing for the gradual introduction of weight-bearing exercises on land. Hydrokinesiotherapy’s effectiveness in improving joint mobility can be attributed to the reduced mechanical loading in the aquatic environment, which facilitates greater range of motion exercises with less pain. The resistance provided by water also offers a low-impact means of strengthening periarticular muscles, which is essential for maintaining joint stability and function. The discussion on joint mobility raises an important consideration regarding the long-term maintenance of these gains. While hydrokinesiotherapy appears to provide substantial improvements in the short term, there is a need for further research to determine whether these benefits are sustained over time and how they compare with long-term outcomes of land-based exercises. Moreover, integrating land-based activities gradually as patients progress might be necessary to ensure that the gains in mobility are maintained when transitioning to regular activities outside the aquatic environment. Quality of life, particularly in terms of physical health, was consistently improved in the hydrokinesiotherapy groups across studies [14,22,23,24,25]. This outcome is particularly noteworthy, as knee OA significantly impacts daily functioning and overall well-being. The improvements in quality of life observed may be a direct result of the combined effects of reduced pain, enhanced mobility, and improved physical function. The holistic nature of hydrokinesiotherapy, which addresses multiple aspects of physical health simultaneously, likely contributes to these positive outcomes. However, it is essential to consider that while physical health improvements are evident, the psychological and social components of quality of life were less frequently assessed in the included studies. Given that chronic conditions like knee OA can have profound effects on mental health and social participation, future studies should incorporate comprehensive measures of quality of life that encompass these dimensions. This would provide a more complete understanding of how hydrokinesiotherapy influences overall patient well-being. The improvements in physical function reported in the studies are of significant clinical importance, as functional decline is a major concern in patients with knee OA [50,51]. Hydrokinesiotherapy appears to offer distinct advantages in enhancing physical function, particularly in activities that involve balance, coordination, and muscle strength. The resistance of water provides a safe environment for performing functional exercises that might be too painful or difficult on land. These findings support the integration of hydrokinesiotherapy into rehabilitation programs for knee OA, particularly for patients who may have difficulty engaging in land-based exercises due to pain or fear of injury [50,52]. However, a key consideration for clinical practice is the transition from aquatic to land-based activities. While hydrokinesiotherapy can significantly improve function in the short term, the long-term goal should be to ensure that patients can maintain these improvements when performing daily activities on land. While the short-term benefits of hydrokinesiotherapy are evident, the long-term sustainability of these outcomes remains underexplored. Future studies should focus on whether improvements in pain and function persist as patients transition to land-based exercises, particularly in terms of long-term joint stability and overall mobility. This scoping review highlights the breadth of evidence on hydrokinesiotherapy for knee OA, particularly from RCTs. However, gaps remain in understanding the long-term effects and comparative efficacy of this treatment. Future research should address these gaps by conducting longer-term follow-ups and comparing hydrokinesiotherapy to other modalities in diverse patient populations. Therefore, a progressive transition plan that gradually increases the intensity and complexity of land-based exercises is recommended. Despite the promising results, this review also highlights several limitations that should be addressed in future research. The heterogeneity of the studies, particularly in terms of intervention protocols, outcome measures, and patient characteristics, poses challenges in comparing results directly. Additionally, the relatively short duration of interventions in most studies raises questions about the long-term sustainability of the benefits observed. Longitudinal studies with extended follow-up periods are needed to determine the durability of hydrokinesiotherapy’s effects. Moreover, while the review focused on key outcomes such as pain, mobility, quality of life, and function, other important factors, such as the psychological impact of hydrokinesiotherapy and patient adherence to exercise programs, were less frequently reported. Future research should aim to include these aspects to provide a more holistic understanding of the benefits and challenges associated with hydrokinesiotherapy. Lastly, the feasibility and accessibility of hydrokinesiotherapy in various healthcare settings should be considered. While the therapeutic benefits are clear, access to facilities equipped for aquatic therapy may be limited, particularly in rural or resource-constrained settings. Research into alternative methods of delivering aquatic therapy, such as home-based aquatic exercises or virtual programs, could expand the accessibility of this effective intervention. Hydrokinesiotherapy represents a highly effective intervention for managing knee osteoarthritis, offering significant improvements in pain, joint mobility, quality of life, and physical function. While the results of this review are encouraging, further research is needed to address the limitations identified and to explore the long-term benefits and practical implementation of hydrokinesiotherapy in diverse patient populations. Integrating hydrokinesiotherapy into standard care practices for knee OA has the potential to enhance patient outcomes and improve overall quality of life.

### Clinical Practice Implications

Hydrokinesiotherapy should be considered a valuable addition to the rehabilitation protocols for knee osteoarthritis, particularly for patients who experience significant pain or mobility limitations with land-based exercises. The buoyancy and warmth of water allow for low-impact, pain-relieving exercises that can enhance joint mobility and improve physical function. Clinicians should integrate hydrokinesiotherapy early in the treatment plan, especially for patients with severe symptoms, and gradually transition to land-based exercises as improvements are made. Accessibility remains a challenge, so exploring partnerships with local aquatic centers or developing home-based aquatic programs could broaden its availability. Hydrokinesiotherapy may be particularly beneficial when initiated in patients with moderate to severe OA symptoms, especially those who struggle with weight-bearing exercises due to pain or reduced mobility. Clinicians should consider delaying hydrokinesiotherapy in patients with contraindications such as active skin infections or severe cardiac conditions.

## 5. Conclusions

Hydrokinesiotherapy shows potential as an effective intervention for managing knee osteoarthritis, offering significant improvements in pain, mobility, and quality of life. Hydrokinesiotherapy is an effective intervention for managing knee osteoarthritis, offering significant benefits in pain reduction, joint mobility, quality of life, and physical function. It should be integrated into treatment plans, particularly for patients who struggle with land-based exercises. Future research should focus on larger randomized controlled trials with extended follow-up periods to assess the long-term benefits of hydrokinesiotherapy, as well as direct comparisons with other therapeutic modalities. While promising, further research is needed to confirm long-term benefits and improve accessibility, ensuring that more patients can benefit from this therapeutic approach.

## Figures and Tables

**Table 1:** Main characteristics of included studies. This table summarizes the characteristics of the randomized controlled trials (RCTs) included in the review, highlighting the study design, language of publication, baseline population, intervention and control groups, outcomes, and outcome measures.

Author(s)	Title	Year	Methods	Results	Outcomes Achieved
Foley et al. [22]	Does hydrotherapy improve strength and physical function in patients with osteoarthritis—a randomized controlled trial comparing a gym-based and a hydrotherapy-based strengthening program	2003	RCT with 105 participants randomized into three groups (hydrotherapy, gym, control); 6-week intervention with 3 sessions per week.	Both gym and hydrotherapy groups showed improvement in quadriceps strength. Hydrotherapy improved SF-12 physical component score.	Increased muscle strength, improved physical function, reduction in pain.
Azizi et al. [14]	Randomized controlled trial of aquatic exercise for treatment of knee osteoarthritis in elderly people	2019	RCT with 31 male participants aged ≥60. Two groups (aquatic exercise, control); 8-week intervention with 3 sessions per week.	Aquatic exercise group showed significant reduction in pain and improvement in static and dynamic balance.	Reduction in pain, improvement in balance and gait parameters.
Taglietti et al. [23]	Effectiveness of aquatic exercises compared to patient-education on health status in individuals with knee osteoarthritis: A randomized controlled trial	2018	RCT with 60 participants aged 60–85. Two groups (aquatic exercises, patient education); 8-week intervention with 2 sessions per week.	Aquatic exercises led to significant improvement in pain and WOMAC scores. No significant difference in functional mobility.	Improvement in pain, quality of life, and WOMAC scores.
Dias et al. [24]	Hydrotherapy improves pain and function in older women with knee osteoarthritis: a randomized controlled trial	2017	RCT with 73 women aged ≥65. Two groups (hydrotherapy, control); 6-week intervention with 2 sessions per week.	Hydrotherapy group showed significant improvements in pain, function, and muscle strength compared to control.	Reduction in pain, improvement in function and muscle strength.
Khruakhorn et al. [25]	Effects of hydrotherapy and land-based exercise on mobility and quality of life in patients with knee osteoarthritis: a randomized control trial	2021	RCT with 34 participants aged 45–75. Two groups (hydrotherapy, land-based exercise). 6-week intervention with 3 sessions per week.	Both interventions improved mobility and quality of life, with significant improvements in SCT for hydrotherapy group.	Improved mobility, quality of life, and reduced pain.

Legend: OA: osteoarthritis, RCT: randomized controlled trial, SCT: stair climb test, SF-12: 12-Item Short Form Health Survey, WOMAC: Western Ontario and McMaster Universities Osteoarthritis Index.

**Table 2:** Baseline Population Characteristics. This table summarizes the baseline characteristics of the populations involved in five key studies evaluating the effectiveness of hydrokinesiotherapy for knee osteoarthritis. It includes details on sample size, age, gender distribution, and the specific inclusion and exclusion criteria applied in each study.

Author(s)	Sample Size	Age (Mean or Range)	Gender Distribution	Inclusion Criteria	Exclusion Criteria
Foley et al. [22]	105 participants	≥50 years (Mean age not specified)	52 females, 53 males	Independence in mobility, no recent physiotherapy or hydrotherapy	Recent knee surgery, planned knee surgery, recent physiotherapy
Azizi et al. [14]	31 participants	≥60 years (Mean age not specified)	All males	Chronic knee pain ≥3 months, radiographic evidence of OA (grades 2 or 3)	Intra-articular injection within 3 months, oral anti-inflammatories within a week, BMI > 34 kg/m^2^
Taglietti et al. [23]	60 participants	60–85 years (Mean age not specified)	Gender not specified	Competence in physical and cognitive abilities, radiographic evidence of OA	History of neurological or orthopedic surgery, severe obesity (BMI > 40 kg/m^2^)
Dias et al. [24]	73 participants	≥65 years (Mean age not specified)	All females	Clinical and radiographic diagnosis of knee OA, no recent trauma or surgery	Recent lower limb trauma, use of walking aids, cognitive impairment
Khruakhorn et al. [25]	34 participants	45–75 years (Mean age not specified)	91.18% females, 8.82% males	Kellgren-Lawrence grade 2–3, independence in mobility	Cardiovascular diseases, rheumatoid arthritis, high uncontrolled blood pressure

**Table 3:** Intervention Characteristics. This table outlines the intervention characteristics of five key studies assessing the effectiveness of hydrokinesiotherapy for knee osteoarthritis. It includes details on the type of intervention, session frequency, duration per session, total intervention duration, and the exercise intensity applied in each study.

Author(s)	Intervention Type	Session Frequency	Duration per Session	Total Intervention Duration	Exercise Intensity
Foley et al. [22]	Hydrotherapy vs. gym-based exercise vs. control	3 sessions per week	30 min	6 weeks	Progressive resistance training
Azizi et al. [14]	Aquatic exercise vs. control	3 sessions per week	60 min	8 weeks	Moderate intensity, following AGS guidelines
Taglietti et al. [23]	Aquatic exercise vs. patient education	2 sessions per week	60 min	8 weeks	Progressive intensity, stretching, dynamic and isometric exercises
Dias et al. [24]	Hydrotherapy vs. control	2 sessions per week	Duration not specified	6 weeks	Moderate intensity, monitored with Borg scale
Khruakhorn et al. [25]	Hydrotherapy vs. land-based exercise	3 sessions per week	45–60 min	6 weeks	Progressive resistance training with speed variations

**Table 4:** Quality Assessment using PEDro and RoB-2 Scales. This table summarizes the quality assessment of the included randomized controlled trials (RCTs) using the PEDro and RoB-2 scales, detailing the methodological rigor and risk of bias.

Author(s)	PEDro Score (Out of 10)	RoB-2 Overall Risk of Bias	RoB-2 Domains Assessed
Foley et al. [22]	8	Low Risk	Randomization Process: Low Risk; Deviations from Intended Interventions: Low Risk; Missing Outcome Data: Low Risk; Measurement of the Outcome: Low Risk; Selection of the Reported Result: Low Risk
Azizi et al. [14]	7	Some Concerns	Randomization Process: Some Concerns; Deviations from Intended Interventions: Low Risk; Missing Outcome Data: Low Risk; Measurement of the Outcome: Low Risk; Selection of the Reported Result: Some Concerns
Taglietti et al. [23]	6	High Risk	Randomization Process: High Risk; Deviations from Intended Interventions: Some Concerns; Missing Outcome Data: Low Risk; Measurement of the Outcome: High Risk; Selection of the Reported Result: Some Concerns
Dias et al. [24]	9	Low Risk	Randomization Process: Low Risk; Deviations from Intended Interventions: Low Risk; Missing Outcome Data: Low Risk; Measurement of the Outcome: Low Risk; Selection of the Reported Result: Low Risk
Khruakhorn et al. [25]	7	Some Concerns	Randomization Process: Some Concerns; Deviations from Intended Interventions: Some Concerns; Missing Outcome Data: Low Risk; Measurement of the Outcome: Low Risk; Selection of the Reported Result: Some Concerns

Legend: PEDro Score: Physiotherapy Evidence Database Score, RoB-2: Risk of Bias 2 Tool.
